# Functional Interactions between the *erupted/tsg101* Growth Suppressor Gene and the *DaPKC* and *rbf1* Genes in *Drosophila* Imaginal Disc Tumors

**DOI:** 10.1371/journal.pone.0007039

**Published:** 2009-09-29

**Authors:** M. Melissa Gilbert, Brian S. Robinson, Kenneth H. Moberg

**Affiliations:** Department of Cell Biology, Emory University School of Medicine, Atlanta, Georgia, United States of America; Institut Pasteur, France

## Abstract

**Background:**

The *Drosophila* gene *erupted* (*ept*) encodes the fly homolog of human Tumor Susceptibility Gene-101 (TSG101), which functions as part of the conserved ESCRT-1 complex to facilitate the movement of cargoes through the endolysosomal pathway. Loss of *ept* or other genes that encode components of the endocytic machinery (e.g. *synatxin7/avalanche*, *rab5*, and *vps25*) produces disorganized overgrowth of imaginal disc tissue. Excess cell division is postulated to be a primary cause of these ‘neoplastic’ phenotypes, but the autonomous effect of these mutations on cell cycle control has not been examined.

**Principal Findings:**

Here we show that disc cells lacking *ept* function display an altered cell cycle profile indicative of deregulated progression through the G1-to-S phase transition and express reduced levels of the tumor suppressor ortholog and G1/S inhibitor Rbf1. Genetic reductions of the *Drosophila* aPKC kinase (DaPKC), which has been shown to promote tumor growth in other fly tumor models, prevent both the *ept* neoplastic phenotype and the reduction in Rbf1 levels that otherwise occurs in clones of *ept* mutant cells; this effect is coincident with changes in localization of Notch and Crumbs, two proteins whose sorting is altered in *ept* mutant cells. The effect on Rbf1 can also be blocked by removal of the γ-secretase component *presenilin*, suggesting that cleavage of a γ-secretase target influences Rbf1 levels in *ept* mutant cells. Expression of exogenous *rbf1* completely ablates *ept* mutant eye tissues but only mildly affects the development of discs composed of cells with wild type *ept*.

**Conclusions:**

Together, these data show that loss of *ept* alters nuclear cell cycle control in developing imaginal discs and identify the *DaPKC*, *presenilin*, and *rbf1* genes as modifiers of molecular and cellular phenotypes that result from loss of *ept*.

## Introduction

Genetic screens in *Drosophila* have identified a relatively small group of mutations that disrupt normal epithelial architecture and lead to neoplastic overgrowth of developing larval imaginal discs, a set of polarized epithelial tissues that grow during larval stages and develop into the majority of adult structures [reviewed in 1]. The genes affected by these mutations encode proteins with conserved human homologs and fall generally into two functional classes: those involved in the establishment and maintenance of apicobasal polarity [reviewed in 2], and those involved in vesicular trafficking of transmembrane proteins [3]–[8]. Genes in this latter group have been termed ‘endocytic tumor suppressor genes’ and include *rab5*, *syntaxin-7/avalanche* (*syx7*/*avl*), *erupted/tumor susceptibility gene-101* (*ept/tsg101* and referred to hereafter as *ept*), and *vps25*. Each of these genes is required at distinct steps in the trafficking proteins from the apical membrane to the lysosome. The latter two genes, *ept* and *vps25*, respectively encode components of the ESCRT (endosomal sorting complex required for transport)-I and ESCRT-II complexes which promote maturation of late-endosomes into multi-vesicular bodies (MVBs) prior to subsequent fusion with the lysosome [9], [10].

Though it is assumed that mutations in these vesicular trafficking factors promote tissue growth in part by removing developmental blocks to excess cell division, there is little direct evidence that links *ept*, *vps25*, or *syx7/avl* mutations to specific cell cycle transitions or to core components of the nuclear cell cycle machinery. Mutations in *ept* are known to block the trafficking and degradation of certain apically localized trans-membrane proteins, including the apical membrane determinant Crumbs and the transmembrane receptor Notch [3], [5], [6], [8], but the effects these molecules have on the cell division process in *ept* mutant cells is not known. Notch has many context-specific links to the cell cycle including controlling levels of the mitotic regulator Cyclin A [11], activity of the dE2f1 transcription factor [11], [12], and expression of the *dacapo*, *string*, and *fizzy related* genes in ovarian follicle cells [13], [14]. Notch has also been reported to collaborate with chromatin modifying factors to silence expression of the *rbf1* gene in eye imaginal disc tumors [15]. Thus, there are many pathways through which Notch could potentially affect either the G2/M or G1/S cell cycle transitions in *ept* mutant cells. The ability of *crb* overexpression to drive imaginal disc neoplasia [4] argues that Crb can also directly or indirectly affect the cell division process. Yet the potential links between Crb–an integral membrane scaffolding molecule with no known intrinsic signaling activity – and the cell division process are not well understood. *crb* indirectly regulates Notch in the larval wing by modulating activity of the γ-secretase complex [16]. However, since mechanisms that deregulate cell division in endocytic tumor suppressor mutants are poorly understood, it is difficult to discern specific pathways through which Notch, Crb, and the myriad of other receptors that are candidate targets of the ESCRT pathway (for example those shown to be affected by loss of the *hrs* gene [17]) might exert pro-proliferative effects in these mutant backgrounds.

We have taken a dual approach to examine cell division control in *ept* mutant eye-antennal tumors: we have sought to identify genetic manipulations that suppress *ept* tumor growth, and in parallel we have characterized the effect of *ept* loss on cell cycle phasing and expression of core cell cycle regulatory factors. We have found that genetic reduction of the DaPKC apical-membrane kinase effectively suppress the growth of *ept* mutant eye-antennal tumors. In parallel, we have found that *ept* mutant eye and wing imaginal discs are enriched for cells in the G2/M phase and depleted for those G1 phase, and this correlates with reduced expression of the nuclear S-phase inhibitor and tumor suppressor homolog Rbf1. These two phenotypes are linked by the observation that expression of a dominant-negative DaPKC transgene (*DN-DaPKC*) in *ept* eye cells is sufficient to prevent the reduction in Rbf1 levels. It is also sufficient to prevent high-level expression of the Upd protein, which is induced in a Notch-dependent manner in *vps25* mutant cells [3]. A similar rescue of Rbf1 levels is observed following removal of the *presenilin* gene (*psn*) from *ept* mutant clones, indicating that cleavage of a Psn substrate(s) contributes to the effect of the *ept* genotype on Rbf1 levels. To test the physiologic significance of the Rbf1 reduction in *ept* cells, we have re-expressed exogenous *rbf1* in either wild type or *ept* mutant eye-antennal tissue and found that excess *rbf1* is able to completely ablate mutant tissue while having little effect on normal tissue. These data indicate that *DaPKC*- and *psn*-dependent loss of Rbf1 from *ept* cells may be a significant factor in their overgrowth.

## Results

### *DaPKC* is required for *ept* tumor growth

**Figure 1:**
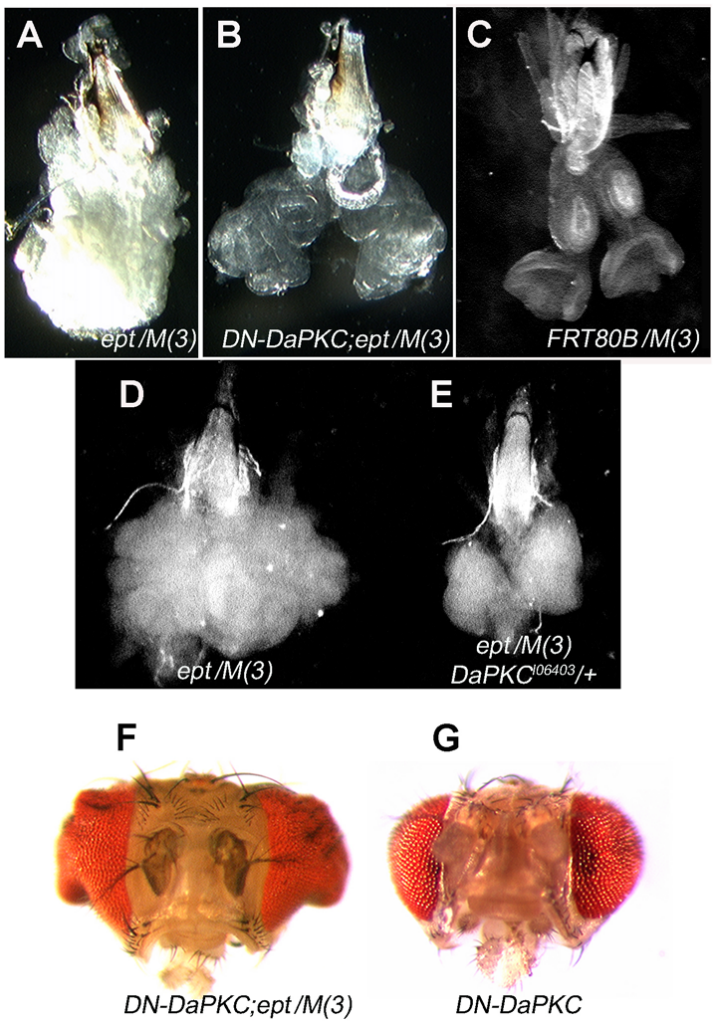
*DaPKC* is required for *ept* tumor growth. Bright-field images of *ept/M(3)* mutant (A), *ept/M(3)* mutant expressing *DN-DaPKC* (B), or *FRT80B/M(3)* (C) eye-antennal discs dissected from wandering 3^rd^ instar larvae. (D,E) Side-by-side image of an *ept/M(3)* mutant eye antennal disc and an *ept/M(3)* disc that is heterozygous for the *DaPKC^k06403^* allele. (F,G) Heads from surviving *ept/M(3)+DN-DaPKC* adults or *DN-aPKC* control animals. Grouped images are to scale.

Under normal circumstances, *ept* mutant eye-antennal tumors created using the cell-lethal *Minute* (*M*) technique (the genotype *eyFLP;;ept^2^,FRT80B/P[m-w+]RpL14^1^,FRT80B* is hereafter referred to as *ept/M(3*)) grow into large unstructured masses (Fig. 1A) that fail to differentiate into recognizable eye tissue and kill the animal bearing them during the late larval and pupal phases [6]. To test the genetic requirements of this tumor-like phenotype, we screened a small collection of alleles of signaling, polarity, and growth regulatory genes (*stat92E*, *crb, lgl, Drosophila aPKC, yki*, *cyclinD*, *dMyc*, *s6k* and others) for their ability to suppress size and/or architectural phenotypes associated with loss of *ept/tsg101*. Alleles of two of these genes had significant effects on the morphology of *ept/M(3)* tumors: the *DaPKC* gene, which encodes an apical-membrane kinase that controls epithelial polarity and endocytosis [18]–[21], and the *stat92E* gene, which encodes the sole fly homolog of the Stat family of mammalian transcription factors [22], [23]. Analysis of the effect of *DaPKC* on *ept* tumor growth is presented here. Expression of a transgene encoding a dominant-negative version of the *DaPKC* kinase (*DN-DaPKC*; [24]) in *ept/M(3)* mutant discs caused these tissues to develop as enlarged eye/antennal structures (Fig. 1B) that are morphologically similar to normal eye discs (Fig. 1C) and contain differentiated photoreceptor neurons (data not shown). Most animals bearing these *DN-DaPKC;ept/M(3)* discs survive to late-pupal and pharate adult stages well beyond the point where animals bearing *ept/M(3)* tumors normally die; a few survive to eclosion and emerge with irregular heads and eyes (Fig. 1F) that are enlarged relative to those expressing the *DN-DaPKC* transgene alone (Fig. 1G). To confirm that this genetic interaction is not an artifact of the *DN-DaPKC* transgene, *ept/M(3)* tumors were also generated in a background heterozygous for the genomic *DaPKC^k06403^* loss-of-function allele [25]. This *DaPKC^k06403^/+* genotype also shrank the size of *ept/M(3)* tumors (Figs. 1D,E), confirming that DaPKC is required for the *ept* tumor phenotype.

### *ept* mutant cells exhibit G1/S cell cycle deregulation

**Figure 2:**
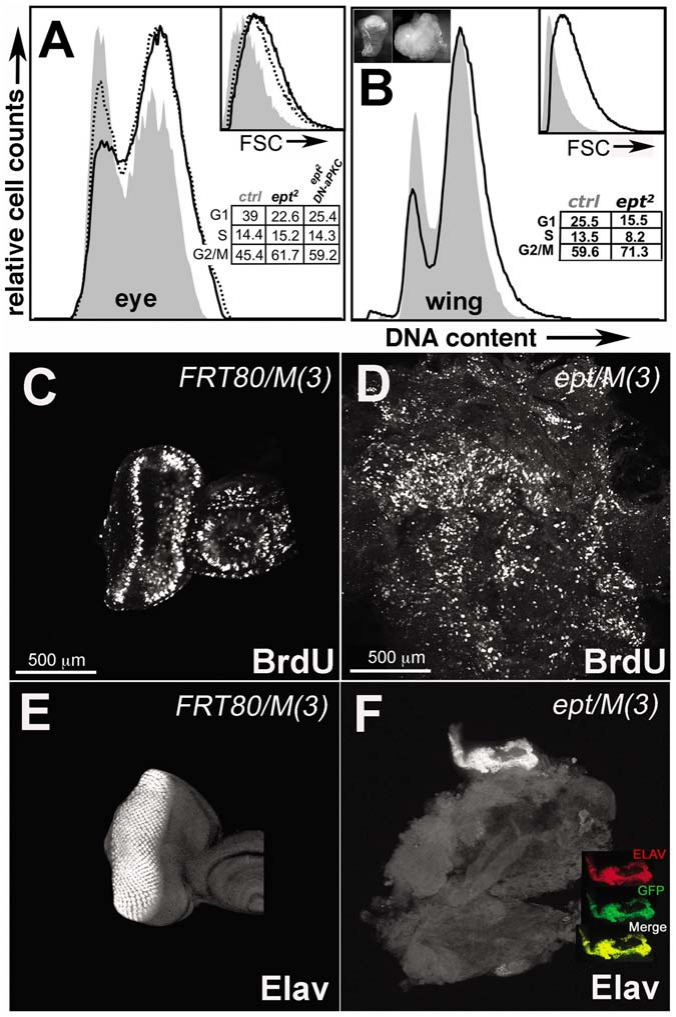
Cell cycle deregulation in *ept* mutant imaginal discs. (A–B) Flow cytometric analysis of cells in control *FRT80B/M(3)* (grey fill), *ept* mutant (black line), or *DN-DaPKC,ept/M(3)* (dotted line) wing or eye imaginal discs. Percent of cells in each cell cycle phase is indicated. Inset images in (B) show a normal wing disc and one composed of cells homozygous for the *ept^2^* allele. Forward scatter (FSC) plot of cell size is also included for each sample. Patterns of BrdU incorporation (C–D) and Elav expression (E–F) in control *FRT80B/M(3)* (C,E) and *ept* mutant (D,F) eye-antennal disc tumors dissected from wandering-stage larvae. Inset in (F) shows expression of Elav (red) only in surviving GFP-positive *Min/+* cells (green).

Because excess cell proliferation is a key factor in tissue hypertrophy, we examined the effect of *ept* loss on the cell cycling properties of cells in eye-antennal discs and whether *DaPKC* might influence this effect. Fluorescence-activated cell sorting (FACS) analysis of *ept/M(3)* eye-antennal discs shows that the population of cells within them is under-represented for 2N G1-phase cells and enriched for S- and G2/M-phase cells relative to control *FRT/M(3)* discs (Fig. 2A). Cell size is also increased in *ept/M(3)* tumors relative to control cells (Fig. 2A, see inset). *ept/M(3)* eye-antennal discs show widespread BrdU incorporation relative to control discs (Figs. 2C–D) and lack cells that express the neuronal marker Elav (Figs. 2E–F). FACS analysis of *ept* mutant wing disc tumors [genotype *UbxFlp;;M(3),FRT80B/ept^2^,FRT80B*] show similar cell cycle and cell size shifts as *ept/M(3)* eye-antennal disc cells (Fig. 2B), although the G2/M-shift is less pronounced in the wing. Thus cell cycling changes associated with loss of *ept* function are not solely due to a block in progression of the eye-specific morphogenetic furrow [26], indicating that loss of *ept* affects G1/S progression in multiple larval discs.

Expression of *DN-DaPKC* in the background of *ept/M(3)* eye-antennal discs led to a reproducible shift of a fraction of cells back into the G1-phase (dotted line, Fig. 2C). The cell cycle shift induced by the *DN-DaPKC* transgene only partially restored cell cycle phasing and had no discernable effect on the enlarged size of *ept* cells, suggesting that additional factors contribute to each of these phenotypes. Consistent with this hypothesis, a reduction in the dose of the *stat92E* gene affects both cell cycle phasing and cell size in *ept/M(3)* tumors (see accompanying paper by Gilbert et al.).

**Figure 3:**
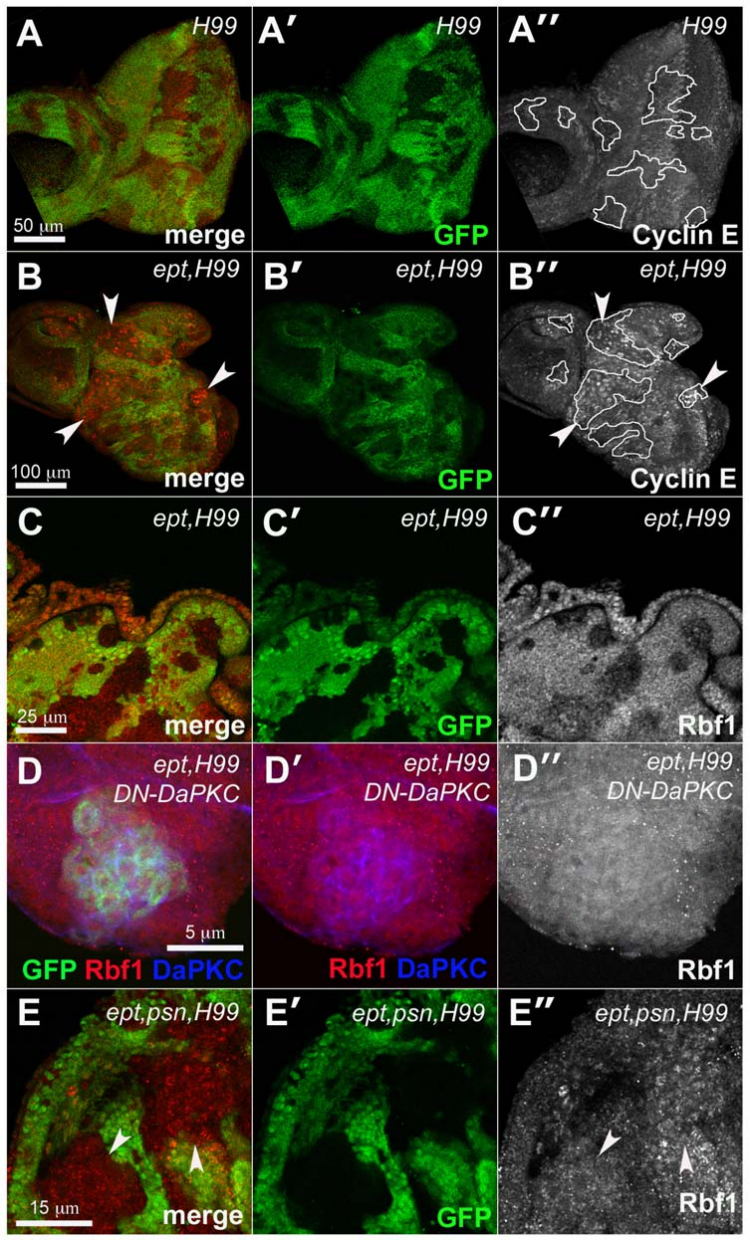
*ept* mutations reduce levels of the Drosophila Rb ortholog Rbf1. Confocal images of larval eye discs containing clones of *H99* mutant cells (A–A″), *ept,H99* double-mutant cells (B–C″) or *ept,H99,psn* triple mutant cells (E–E″) marked by the absence of GFP (green) co-stained for CycE (red in A–B″), or Rbf1 (red in C–C″, and E–E″). Tracing in A″ and B″ outlines *H99* and *eptH99* mutant clones respectively. The anti-CycE signal was recorded at the same optical settings in panels A″ and B″. Arrowheads in panels B and B″ denote *ept,H99* double mutant clones that express CycE. (D–D″) MARCM-mediated expression of DN-DaPKC (blue) in *ept,H99* double-mutant cells marked by GFP (green) restores levels of Rbf1 (red). Arrows in panel E denote clones of *ept,H99,psn* cells that express normal levels of Rbf1 relative to adjacent control cells.

We next examined the levels of G1/S regulatory proteins in clones of eye disc cells doubly mutant for *ept* and the *H99* chromosomal deletion, which removes the genes *rpr*, *grim*, and *hid* [27] and prevents activation of the pro-death caspase enzymes [28]–[33] that are otherwise detected at high levels in *ept* mutant clones of cells (Figure S1); their removal thus allows recovery of larger *ept* mutant clones and permits molecular analysis of protein epitopes that might otherwise be degraded. The expression of two key regulators of G1/S, the pro-division protein Cyclin E (CycE) and Rbf1, the *Drosophila* homolog of the retinoblastoma (Rb) tumor suppressor protein [34], were found to be affected by the *ept* genotype. Compared to *H99* control clones (Fig. 3A–A″), some *ept,H99* mutant clones express elevated levels of CycE protein (Fig. 3B–B″, see arrows). CycE is also slightly elevated in some normal cells that surround mutant clones, which is likely a reflection of the previously described non-autonomous mitogenic effect of *ept* mutant cells [6]. Neither of these effects on CycE are not apparent in all clones, and are thus not a strongly penetrant part of the *ept,H99* phenotype. By contrast, immunostaining with a monoclonal antibody specific to Rbf1 [34] shows that levels of this protein are clearly reduced in *ept,H99* disc cells compared to surrounding control cells (Fig. 3C–C″). Clones of *H99* mutant cells do not show the same effect (Figure S2), indicating that *ept* is required to maintain normal levels of Rbf1 protein in eye-antennal disc cells.

### *DaPKC* and *psn* are required for the effect on Rbf1 levels in *ept* mutant cells

Given the effect of the *DaPKC* alleles on *ept/M(3)* tumor growth, we next examined whether *DaPKC* activity might affect Rbf1 levels in *ept* mutant cells. To do this, the *DN-DaPKC* transgene was expressed specifically in *ept,H99* mutant cells using the MARCM technique [35]. Although this led to a significant reduction in the size of *ept,H99* clones (paralleling the effect of the *DN-DaPKC* transgene on *ept* tumor size), close examination of *DN-DaPKC+ept,H99* clones stained with the anti-Rbf1 antibody revealed no obvious difference in Rbf1 levels relative to surrounding normal cells (Fig 3D–D″). By this measure, *DaPKC* activity is required for the effect of the *ept,H99* genotype on Rbf1 levels. Because of the somewhat variable effect of the *ept,H99* genotype on CycE, CycE levels were not examined in this MARCM *DN-DaPKC* background. *DaPKC* is known to regulate a number of cellular processes and pathways ([18] and reviewed in [19]), including the Notch pathway in larval brain neuroblasts [36]. To test whether an allele of a Notch pathway component might also alter the effect of the *ept,H99* genotype on Rbf1, a strong loss-of-function allele of the *presenilin* gene (*psn^227^*;[37]) was recombined onto the *ept,H99* chromosome. *psn* encodes a required component of the γ-secretase that cleaves and releases the Notch intracellular domain and it's activity is needed for Notch activation in vivo [38], [39], Because all three loci are on the left arm of chromosome 3, this *ept,H99,psn* mutant chromosome allows for the production of somatic clones of triple mutant cells. These cells are deficient in *ept* function and Psn activity, but give rise to easily detectable clones due to their inability to die. Immunostaining with the anti-Rbf1 antibody indicates that the level of Rbf1 in these *ept,H99,psn* cells (arrows in Fig. 3E–E″) is similar to that in surrounding normal cells. Thus, loss of *psn* has a similar effect on levels of the anti-Rbf1 epitope in the *ept,H99* genotype as does expression of the *DN-DaPKC* allele.

### Effect of *DN-DaPKC* on Notch and Upd in *ept* cells

**Figure 4:**
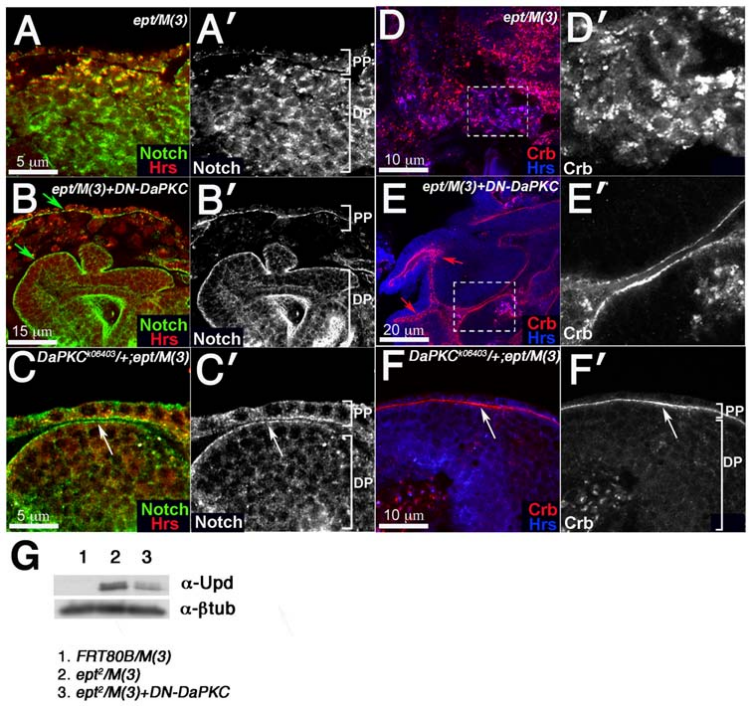
*DaPKC* is required for cytoplasmic accumulation of Crb and Notch in *ept* mutant tissues. Confocal images of *ept/M(3)* (A–A′ and D–D′), *ept/M(3)+DN-DaPKC* (B–B′ and E–E′), or *ept/M(3),DaPKC^k06403^/+* (C–C′ and F–F′) tumors stained for Notch and Hrs (green and red respectively in A–C) or Crb and Hrs (red and blue respectively in D–F). Dashed boxes in panels D and E are enlarged in panels D′ and E′. Arrows in panels B and C mark membrane-associated Notch in disc and peripodial cells. Arrows in panels E and F mark membrane-associated and apical junctional Crb staining. Peripodial cell layer (PP) and disc proper (DP) are labeled in panels A′, B′, C′, and F′ so as to orient the image relative to overall disc structure. Image in panel D′ is of *ept* mutant DP cells; image in E′ is structured lobes of *ept* mutant DP cells that are able to form the presence of the *DN-DaPKC* transgene. (G) Western blot analysis of Upd levels in *FRT80B/M(3)* (lane 1), *ept/M(3)* (lane 2), and *ept/M(3)+DN-DaPKC* (lane 3) larval eye-antennal discs. The blot was stripped and re-probed with an anti-β−gal antibody as a loading control.

The similar effect of the *psn^227^* allele and the *DN-DaPKC* transgene on the Rbf1 epitope in *ept* mutant cells led us to examine Notch protein in *ept* mutant cells in backgrounds in which DaPKC activity is reduced. *ept/M(3)* tumors grow as disorganized masses that lack ‘landmarks’ normally associated with the eye disc (e.g. morphogenetic furrow, optic stalk, eye-antennal boundary, etc) thus depriving the tissue of any A/P or D/V reference points. We have therefore used the sole remaining disc feature, the overlying peripodial membrane, to orient each image. As shown in a prior study [6], loss of *ept* causes the eye disc proper (DP) to grow as disorganized groups of cells surrounded by a layer of peripodial cells (PP) in which the Notch protein shows increased co-staining with the endosomal protein Hrs (Fig. 4A–A′). This co-localization of Notch and Hrs is most apparent in PP cells and in more cortical regions of the DP (upper portion of image in Fig. 4A), suggesting that Notch/Hrs-positive endosomes may accumulate in more apical regions of DP cells. Notch-Hrs co-localization has also been observed in cells lacking the ESCRT-II subunit gene *vps25* [3], [5] and has been interpreted as an indication that Notch normally traffics through ESCRT-I and -II vesicular compartments on its way to the lysosome. Interestingly reducing DaPKC activity, either by expression of the *DN-DaPKC* transgene (Fig. 4B–B′) or heterozygosity for the *DaPKC^k06403^* loss-of-function allele (Fig. 4C–C′), alters the pattern of Notch protein localization in *ept/M(3)*: a larger proportion of the anti-Notch signal is detected on outer surface of the DP and on the apical face of PP cells (arrows in Fig. 4B), with a corresponding drop in the proportion detected in Hrs-positive structures in cytoplasm of cells in the DP. *DaPKC* alleles have a similar effect DP and PP populations of Crumbs (Fig. 4D–F), a protein that is normally trapped in cytoplasmic puncta in *ept* mutant cells [6] and that is known to be regulated by *DaPKC* [24]. As recent studies have suggested that Notch cleavage and activation requires internalization of Notch protein into specific endosomal compartments [40], we sought to determine if changes in Notch localization following expression *DN-DaPKC* might correlate with changes in expression of a validated Notch target. It has previously been shown that expression of a *Notch* RNA interference ‘knock-down’ construct in *vps25* mutant eye disc cells is sufficient to block overproduction of the Unpaired protein (Upd), which is otherwise expressed at very high levels in *vps25* mutant cells [3]. The overexpression of Upd in *ept* mutant cells has also been shown to occur by a Notch-dependent mechanism [6]. We find that expression of the *DN-DaPKC* is sufficient to substantially blunt the elevation in Upd levels as measured by immunoblotting for total Upd protein present in *ept/M(3)* eye-antennal discs (Fig. 4G). Thus, in addition to its effect on *ept/M(3)* tumor growth, Rbf1 levels, and Notch localization, DaPKC is also required for Notch-dependent hyper-accumulation of Upd in *ept/M(3)* tumors.

### Transgenic expression of *rbf1* in *ept/M(3)* tumors

**Figure 5:**
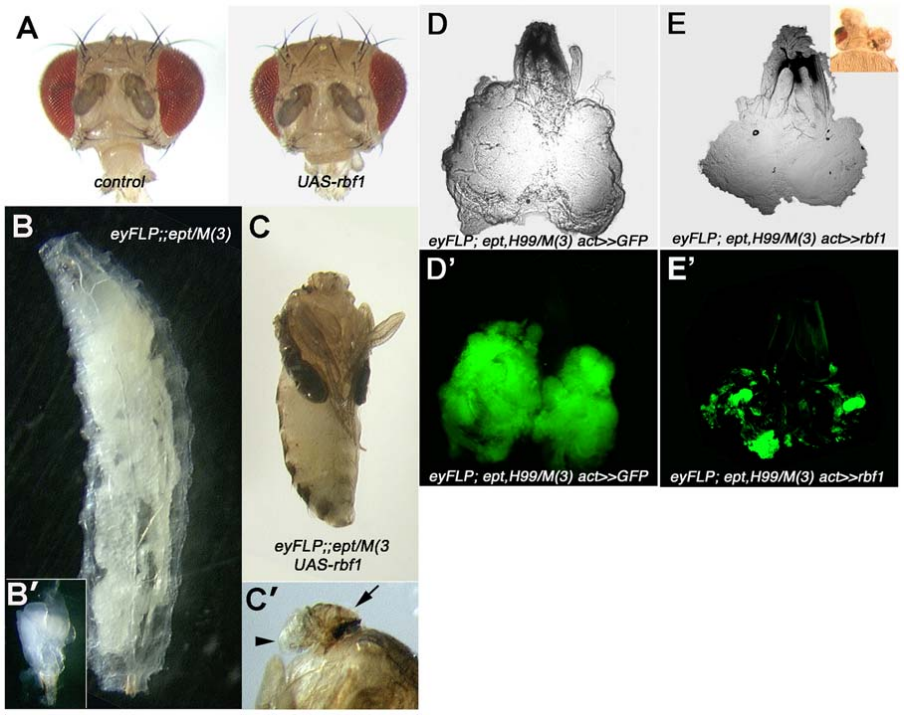
Effect of re-expressing *rbf1* in *ept* mutant eye-antennal discs. (A) Overexpression of *rbf1* in the developing eye and head of control animals results in a mild reduction in adult organ size. Overexpression of *rbf1* in the developing eye and head tissue of animals bearing *ept* eye-antennal tumors blocks the *ept/M(3)* giant larval phenotype (B) and results in headless adults (C) with small maxillary palps and a remnant of head cuticle (indicated by arrowhead and arrow respectively in C′). Inset in B′ shows an *ept* mutant eye-antennal tumor to scale with respect to B and C. Actin ‘Flp-out’ mediated overexpression of *GFP* (D–D′) or *GFP* and *rbf1* (E–E′) in apoptosis-compromised *ept,H99* mutant eye antennal discs reduces clone size (D′ vs E′).

In light of the well-known role Rb family proteins play in regulating the G1-to-S phase cell cycle transition [reviewed in 41], reduced Rbf1 expression in *ept* mutant cells might be predicted to impair the Rbf1-mediated block to unregulated S-phase entry. To test what effect re-expression of *rbf1* might then have on the *ept* tumor phenotype, a *UAS-rbf1* transgene was driven in the background of either normal eye-antennal discs or *ept* mutant eye/antennal tumors. *rbf1* expression in control *FRT80B/M(3)* eyes/heads leads to a moderate reduction in eye and head size (Fig. 5A). In contrast, re-expression of *rbf1* in *ept* eye/antennal tumors completely blocks the growth of the mutant tissue and results in headless pharate adults (Fig. 5B–C). A residual lump of cuticle (arrow in Fig. 5C′) is all that remains of tumors that would otherwise overgrow and kill the animal as an enlarged larva (e.g. Fig. 5B–B″). Expression of *rbf1* thus has a significant and specific effect on the terminal organismal phenotype resulting from *ept* eye-antennal tumors. To test if the effect of *rbf1* on *ept/M(3)* tumor growth is due to enhanced rates of apoptosis, the previous experiment was repeated in *ept,H99/M(3)* tumors that express *UAS-GFP* under control of the *eyFLP* and *Actin5c>CD2>Gal4* transgenes [42], such that each ‘flip-out’ event creates a clone of GFP-positive mutant cells. In the absence of the *UAS-rbf1* transgene, these *ept,H99* mutant tumors are uniformly green (Fig. 5D–D″), indicating that clones of GFP-expressing *ept,H99* mutant cells take over the majority of the organ. However, in the presence of the *UAS-rbf1* transgene, GFP-positive clones of *ept,H99* mutant cells remain quite small (Fig. 5E′), indicating that exogenously produced Rbf1 retains the ability to retard the overgrowth of *ept,H99* mutant cells. At an organ level, re-expression of *rbf1* in the apoptosis-compromised background of *eptH99/M(3)* tumors results in pharate adults with small heads and eyes (inset, Fig. 5E). Thus, the ability of *rbf1* to block *ept* tumor growth is only partly reduced in a background in which cell death is compromised. These observations demonstrate that an eye-antennal disc composed of *ept* mutant cells responds differently to over-expression of *rbf1* than a disc composed of normal cells eye antennal cells, and suggests that re-introducing *rbf1* either slows proliferation of *ept,H99* mutant cells or kills them by an *H99*-independent mechanism; alternatively, specification of early progenitors of the head/eye fate may be defective in cells that simultaneously overexpress *rbf1* and lack *ept*.

## Discussion

The data presented here indicate that the DaPKC kinase plays a significant role in the growth of *ept* mutant imaginal disc tumors. DaPKC is also required for tumor growth in other Drosophila mutants [36], [43]–[45], suggesting that DaPKC may be generally required for excess proliferation in many different backgrounds. The molecular mechanism through which reduced DaPKC activity exerts these effects in *ept/M(3)* cells is not known, although it correlates with increased apical membrane localization of two membrane-bound proteins, Crumbs and Notch, that otherwise aggregate in vesicle-associated structures in the cytoplasm of *ept* mutant cells [6]. DaPKC directly phosphorylates Crumbs and the endocytic adaptor Numb [36], [44], [46], which can in turn inhibit Notch [reviewed in 47]. Thus the effect of reduced DaPKC activity on *ept* tumor growth could be mediated exclusively through effects on Notch and Crb. However two considerations suggest this is unlikely: first, mutations in the *hrs* gene affect the vesicular trafficking of many different receptors [17], suggesting that signals transduced through many receptors may contribute to the overall *ept* tumor phenotype; second, aPKC kinases appear to play a general role in promoting endocytic internalization [18], again suggesting that reduced DaPKC function may affect many different pathways. In light of these considerations, it seems more likely that *DaPKC* alleles partially rescue *ept* phenotypes not because they specifically affect the endocytic uptake of one or two apical-membrane proteins (e.g. Crb and Notch), but because they generally lessen the endocytic uptake of a spectrum of proteins that are otherwise trapped in the late-endosome of *ept* mutant cells. Thus DaPKC might act as a ‘permissive factor’ in *ept* tumor growth via a positive role in endosomal trafficking upstream of ESCRT-1; in its absence a set of proteins that normally enter the endolysosomal pathway and become trapped in *ept* mutant endosomes are shunted toward an alternate fate that precludes their accumulation in late endosomal compartments. Properly testing this model will require a comprehensive analysis of the requirement for DaPKC in sorting individual candidate proteins, and in controlling the activity of specific polarity, signaling, and proliferative pathways in which these proteins act.

The effect of *ept* loss on G1/S control in imaginal discs confirms that one element of the *ept* tumor phenotype is deregulated cell division. Additionally, the data show that this correlates with a requirement for *ept* to maintain normal levels of the key S-phase inhibitor Rbf1 in imaginal disc cells. Loss of Rb homlogs in multiple species is sufficient to accelerate tumor progression [reviewed in 48]. Thus the effect of *ept* on Rbf1 provides a link between a member of the ‘neoplastic’ tumor suppressor genes and an established cell cycle regulator and tumor suppressor protein. Our experiments argue that this effect on Rbf1 is significant to the physiology of *ept* mutant tissues and identify two factors, the DaPKC kinase and the γ-secretase subunit Psn, as required for the reduction of Rbf1 in *ept* mutant cells. *ept* alleles do not affect on Psn protein levels in the eye disc (data not shown), indicating that this requirement for Psn is probably indirect via cleavage of another factor whose activity controls Rbf1 levels. The Notch receptor is a candidate for this role: it is trapped and hyper-activated in *ept* mutant endosomes [6], and its activation mechanism requires a cleavage event that studies have found correlates with Notch endosomal localization [40]. Moreover, ectopic Notch activity has been reported to facilitate transcriptional silencing of the *rbf1* gene in an eye disc tumor model [15]. Thus the effect of the *psn* allele on Rbf1 levels in *ept* cells could indicate a role for Notch upstream of Rbf1. Consistent with this, the ability of the dominant-negative *DaPKC* allele to restore Rbf1 levels in *ept* cells correlates with its ability to block overproduction of the Upd protein, a Notch-target in ESCRT mutant cells. The ability of DaPKC to act as a pro-growth ‘proliferation factor’ in larval brain neuroblasts (NBs) a cell type that uses asymmetric divisions to regulate the fate and proliferative potential of daughter cells, has also been genetically linked to Notch activity [36], [43], [44]. Though these observations suggest that the effect of *DaPKC* and *psn* on Rbf1 levels in *ept* mutant cells may reflect a Notch-regulatory role for both genes, it remains possible that *psn* and *DaPKC* are also involved in a Notch-independent signaling pathway that is responsible for the drop in Rbf1 levels in *ept* mutant disc cells. As certain endocytic tumor suppressors activate Notch (e.g. *ept* and *vps25*) and others do so to a much lesser extent (e.g. *ayx7/avl*), one way to begin to address this question may be to examine the pattern of Rbf1 expression in each of these mutant backgrounds.

As a component of the ESCRT-1 complex, *ept* developmental phenotypes are expected to reflect the combined deregulation of the myriad proteins that traffic through ESCRT-1 dependent compartments of the endolysosomal pathway. Indeed certain elements of the *ept* mutant phenotype (e.g. increased cell size) do not appear to respond to reduced *DaPKC* activity. Many other prominent signaling molecules are mislocalized in *Drosophila* wing disc cells mutant for the endocytic regulator *hrs* [17], which acts at a sorting step prior to the ESCRT-1 complex [49]. The pathways in which these molecules act should thus be considered as additional candidate effectors of *ept* mutations. This expanding array of potential ESCRT targets raises the possibility that inactivation of *ept* will have tissue- and stage-specific phenotypes that reflect the changing pattern of proteins targeted for endolysosomal degradation in various cell types. If targeted endocytosis regulates a similar array of proteins in certain mammalian epithelial cells as it does in *Drosophila* imaginal discs, it may be that defects in this process will act via an aPKC- and Psn-dependent pathway to produce neoplastic phenotypes similar to those observed with alleles of *Drosophila ept*.

## Materials and Methods

### Genetics

Crosses were carried out at 25°C unless otherwise indicated. *DN-DaPKC*-mediated rescue of *ept* tumor phenotypes was optimal at 20°C. *ept* mutant eye clones were generated as described previously [4], [6]. *ept,H99* and *ept,H99,psn* mutant eye clones were generated by crossing either *w;;ept^X1^,Df(3L)H99,FRT80B/TM6B* or *w;;ept^X1^,Df(3L)H99,psn^227^,FRT80B/TM6B* males to *yweyFLP;;P[m-w^+^;ubiGFP]*,*FRT80B* females. *ept* mutant eye-antennal tumors were generated by crossing *w;;ept^2^,FRT80B* males and *yweyFLP;;P[m-w^+^]RpL14^1^,FRT80B/TM6B* females. ‘*DN-DaPKC,ept/M(3)’* animals were generated by crossing the *w;UAS-DN-aPKC;ept^2^,FRT80B/TM6B* stock to the *y,w,eyFLP; act>y^+^>Gal4/CyO:twi-GFP;P[m-w^+^]RpL14^1^,FRT80B/TM6B* stock. Expression of *DN-DaPKC* in *ept,H99* clones was achieved by crossing *w;UAS-DN-DaPKC;ept^X1^,Df(3L)H99,FRT80B/TM6B* males to *eyFLP;tub-Gal4;tub-Gal80,FRT80B* females. ‘*rbf1,ept/M(3)*’ and ‘*rbf1,eptH99/M(3)*’ animals were generated by crossing the *w;UAS-rbf1/CyO;ept^2^,FRT80B/TM6B* or *w;UAS-rbf1/CyO;ept^X1^,Df(3L)H99,FRT80B/TM6B* stocks to the *y,w,eyFLP; act>y^+^>Gal4/CyO:twi-GFP;P[m-w^+^]RpL14^1^,FRT80B/TM6B* stock. The *DaPKC^k06403^* and *psn^227^* were obtained from the Bloomington *Drosophila* Stock Center (BDSC). The *Df(3L)H99* stock was a gift of K. White. The *UAS-DN-DaPKC* chromosome 2 stock was a gift of D. Bilder. *DN-DaPKC*-expressing *ept* mutant clones were generated by crossing the *yweyFLP;tub-Gal4/CyO;tub-Gal80,FRT80B* stock (gift of J. Treisman) to *w;UAS-DN-DaPKC/CyO,twi-GFP;ept^2^,FRT80B/TM6B*.

### Flow Cytometry

Eye or wings discs were dissociated in PBS Trypsin-EDTA and stained with 20 µM DRAQ-5 (Biostatus Limited). Data were acquired on a Becton Dickinson LSR II flow cytometer via a 755 nM Red laser with a 780/60 nM BP collection filter and were analyzed with FACSDiva Software.

### Microscopy & Immunohistochemistry

Immunostaining and confocal microscopy was performed as described previously [50]. Antibodies used were: rat anti-Crb-extra (gift of U. Tepass and E. Knust) 1∶500; guinea pig anti-Hrs (gift of H. Bellen) 1∶1000; mouse anti-BrdU (Becton Dickinson) 1∶100; mouse anti-CycE, 1∶5 (gift of H. Richardson); mouse 9C6 anti-Notch (DSHB) 1∶50; mouse anti-Rbf1 DX5 (gift of W. Du), 1∶50; rat anti-ELAV (DSHB) 1∶1000; goat anti-rabbit Cy5, goat anti-mouse Cy3, goat anti-guinea pig Cy3, and goat anti-rat Cy3 (Jackson Laboratories) each at 1∶50. BrdU incorporation assays were performed as described previously [51].

## Supporting Information

Figure S1 Levels of cleaved Caspase-3 are elevated in *ept/tsg101* mutant eye clones. Clones of *ept/tsg101* mutant cells marked by the absence of GFP (green) contain many dying cells, as indicated by staining for cleaved Caspase-3 (blue).
(1.84 MB TIF)

Figure S2 Rbf1 levels are unaffected by the *H99* deletion. Confocal image of *H99* clones (lacking GFP) in a mosaic 3rd instar eye imaginal disc stained with the anti-Rbf1 antibody (red).
(0.73 MB TIF)
