# Supplementary figures and images for: Functional Interactions between the erupted/tsg101 Growth Suppressor Gene and the DaPKC and rbf1 Genes in Drosophila Imaginal Disc Tumors
Source: PLoS One. 2009 Sep 29;4(9):e7039. doi: 10.1371/journal.pone.0007039 (PMC2739425; doi:10.1371/journal.pone.0007039)

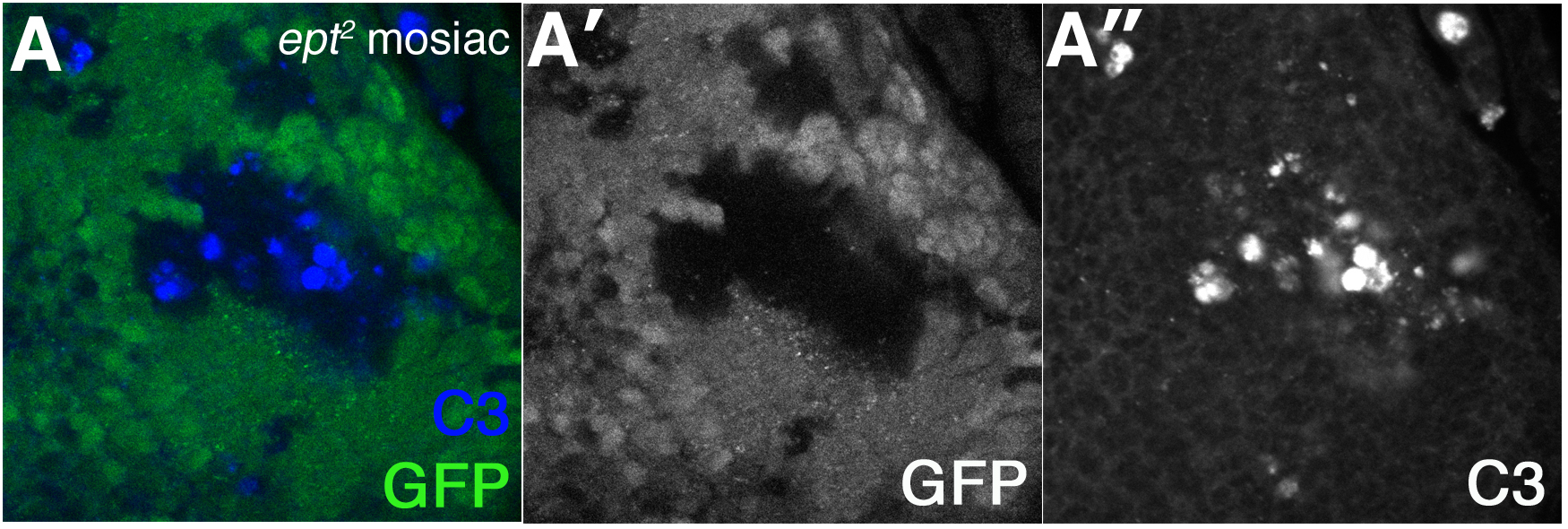

Supplement: Figure S1 — Levels of cleaved Caspase-3 are elevated in ept/tsg101 mutant eye clones. Clones of ept/tsg101 mutant cells marked by the absence of GFP (green) contain many dying cells, as indicated by staining for cleaved Caspase-3 (blue). (1.84 MB TIF) [file pone.0007039.s001.tif]

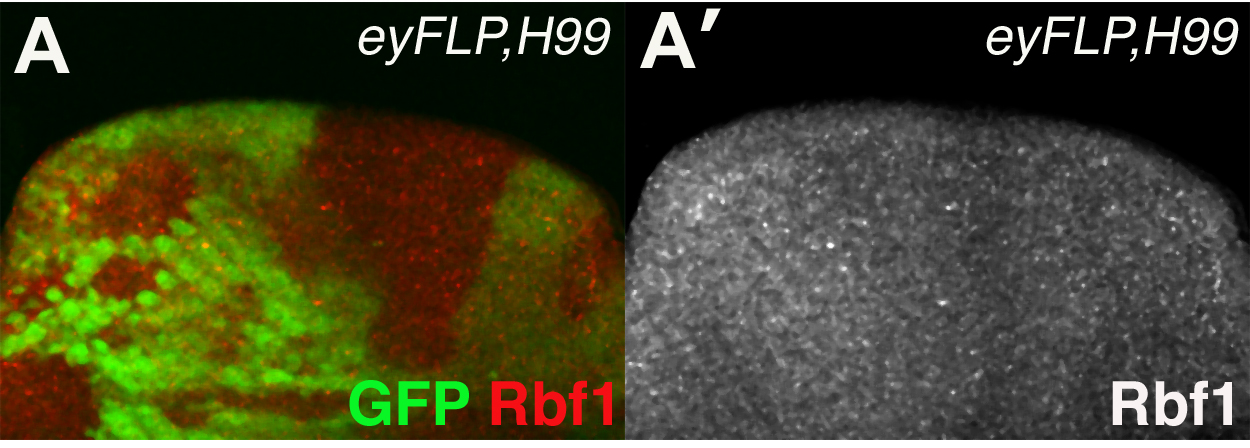

Supplement: Figure S2 — Rbf1 levels are unaffected by the H99 deletion. Confocal image of H99 clones (lacking GFP) in a mosaic 3rd instar eye imaginal disc stained with the anti-Rbf1 antibody (red). (0.73 MB TIF) [file pone.0007039.s002.tif]
